# Effect of Combining a Prebiotic (Autolyzed Yeast from *Saccharomyces cerevisiae*) and Probiotic (*Bacillus subtilis*) Added in a High-Energy Diet on Growth Performance, Dietary Energetics, and Carcass Traits of Fattening Hairy Lambs

**DOI:** 10.3390/ani16040610

**Published:** 2026-02-14

**Authors:** Jesús A. Quezada-Rubio, Alfredo Estrada-Angulo, Beatriz I. Castro-Pérez, Jesús D. Urías-Estrada, Elizama Ponce-Barraza, Lucía de G. Escobedo-Gallegos, Daniel A. Mendoza-Cortez, Alberto Barreras, Octavio Carrillo-Muro, Alejandro Plascencia

**Affiliations:** 1 Faculty of Veterinary Medicine and Zootechnics, Autonomous University of Sinaloa, Culiacan 80260, Mexico; jesus.quezada.fmvz18@uas.edu.mx (J.A.Q.-R.); alfred_vet@uas.edu.mx (A.E.-A.); laisa_29@hotmail.com (B.I.C.-P.); david.urias@uas.edu.mx (J.D.U.-E.); elizama.ponce@hotmail.com (E.P.-B.); lucia.escobedo@uabc.edu.mx (L.d.G.E.-G.); danielmendoza@outlook.com (D.A.M.-C.); 2 Veterinary Science Research Institute, Autonomous University of Baja California, Mexicali 21100, Mexico; beto_barreras@yahoo.com; 3 Academic Unit of Veterinary Medicine and Zootechnics, Autonomous University of Zacatecas, General Enrique Estrada 98500, Mexico; octavio_cm@uaz.edu.mx

**Keywords:** symbiotic, fattening lambs, performance, dietary energetics, carcass, feeding strategy

## Abstract

Due to their specific properties, the autolyzed yeast *Saccharomyces cerevisiae* (SC) and bacterial *Bacillus subtilis* (BS) theoretically can have a synergistic effect when combined and offered in ruminant diets. Thus, the aim of this experiment was to evaluate the effect of their combination as feed additives on growth performance, dietary energy, carcass traits, and visceral organ mass in finishing lambs. For this reason, 48 Pelibuey × Katahdin lambs (98 ± 17 d age; initial weight = 20.25 ± 3.37 kg) were used in a feeding trial lasting 83 d. Lambs were blocked by weight and assigned to 24 pens. Treatment consisted in supplementing a high-energy diet with probiotic and/or prebiotic as follows: (1) finishing diet without probiotic or prebiotic supplementation (Control), (2) finishing diet supplemented with 1.5 g SC/kg diet, (3) finishing diet supplemented with 1.5 g BS/kg diet, and (4) finishing diet supplemented with 1.5 g SC plus 1.5 g BS/kg diet. There were no synergistic (interaction) effects by combining SC + BS in any of the variables evaluated. Lambs that were supplemented with BS showed a very similar response on dry matter intake (DMI, *p* = 0.41), average daily gain (ADG, *p* = 0.64), carcass traits (*p* ≥ 0.08), tissue composition (*p* ≥ 0.32), and relative visceral organ mass (g/kg EBW, *p* ≥ 0.15) than non-supplemented lambs. Compared to the control group, lambs that received SC alone or in combination with BS showed greater average daily gain (12.0%, *p* = 0.03), gain efficiency (6.1%, *p* = 0.04) and observed-to expected dietary energy efficiency (5.5%, *p* = 0.04). Supplemental SC and SC + BS increased hot carcass weight (*p* = 0.04) without effects on the rest of the variables evaluated including the shoulder tissue composition whole cuts, and visceral organ mass. It was concluded that SC improves growth performance and dietary energy in finishing lambs without changes in carcass traits or carcass composition. Combining SC with BS did not improve the magnitude of the response of SC supplemented alone. In this study, the inclusion of a 1.5 g/kg diet of BS during a long-term period (83 d) did not show benefits to finishing lambs.

## Simple Summary

Due to their specific properties, the prebiotic autolyzed yeast *Saccharomyces cerevisiae* (SC) and the probiotic bacterium *Bacillus subtilis* (BS) can theoretically exhibit a synergistic effect when combined and offered in ruminant diets. Thus, the aim of this experiment was to evaluate the combination of both as feed additives on feed utilization efficiency and carcass traits in fattening lambs. For that, SC and BS were offered alone or in combination for 83 days, and one group served as the control (non-additive supplementation). Lambs that received SC showed improvements of 12% in growth rate, 6.1% in gain efficiency, and 5.5% in observed-to-expected dietary energy utilization, without altering carcass traits or composition. Contrary to our expectations, combining autolyzed SC with BS did not enhance those responses when compared to autolyzed SC alone. On the other hand, lambs that received BS supplementation alone did not show any improvements when compared to non-supplemented lambs.

## 1. Introduction

In several countries around the world, antibiotics are still used as growth promoters or feed efficiency improvers in ruminant fattening systems. However, there is growing concern about the elimination of these products for economic and health reasons [1]. Thus, the search for safe alternatives for the growth and/or feed efficiency improvers has been increasing. There is a wide variety of alternatives that have been tested as feed additives in ruminant fattening, of which probiotics and prebiotics are possibly the most popular [2,3]. Yeasts can act as probiotics (live yeast) or prebiotics (autolyzed yeast) supplements, which modulate intestinal flora through a competitive process with intestinal pathogens. As well as altering rumen fermentation and promoting gastrointestinal (GIT) health analogous to antibiotics [4], probiotics also have antioxidant and immunological effects [5]. All of these effects are associated with an increase in animal growth rate and/or feed efficiency in broilers [6], pigs [7], cattle [8], and lambs [9,10]. One of the probiotics commonly used as a feed additive in livestock is *Bacillus subtilis* (BS, a Gram-positive bacterium), which is unique in its ability to form spores, and because it is a facultative aerobe, it can survive under adverse conditions, as well as being able to form robust protective biofilms against pathogenic agents [11]. These characteristics could make BS more effective at the lower tract level than other widely used probiotics (e.g., *Saccharomyces cerevisiae*). In this manner, BS has been successfully used as a feed additive to improve growth performance in pigs [12,13,14] and poultry [15,16,17], although the positive growth performance effects seem to be evident primarily when pigs and broilers receive BS supplement under disease challenge conditions. Surprisingly, the impact of BS supplementation on ruminants on parameters such as ruminal fermentation, nutrient digestion, and productive performance has been very modest [8,18,19]. Likewise, Estrada-Angulo et al. [20] did not observe improvements on ADG, feed efficiency, or in dietary energy utilization when lambs were supplemented with 2 g BS (2.2 × 10^8^ CFU)/kg diet; however, BS showed a synergistic effect, enhancing growth performance parameters when it was supplemented in combination with the antibiotic monensin (a ruminal fermentation modifier).

The yeast *Saccharomyces cerevisiae* (SC) alters ruminal bacterial communities and rumen fermentation and promotes GIT health analogous to the antibiotic monensin [21]. The main effects of yeast SC are that scavenge oxygen within the rumen, creating a more anaerobic environment, which is required by ruminal microorganisms implicated in nutrient degradation, microbial N synthesis, and VFA production [22]. Furthermore, SC is believed to provide growth factors to the rumen microorganisms, including organic acids, B vitamins, and amino acids [23], stimulating the growth of lactate-utilizing bacteria and protozoal population [24]. All these effects of SC improving the energy utilization from feed increase productivity and health. Despite the autolyzed yeast’s inability to grow in rumen fluid, it retains its metabolic activity and viability, although this theoretically would limit its benefits only to the ruminal environment, with very little impact on the lower tract [25]. Even when the mechanism effects of probiotics and prebiotics are not fully understood, it has been demonstrated that, depending on source (yeast or bacterial, strain, among others), they act in different ways in GIT. In this manner, because of its different natures can enhance animal productivity and health by complementary effects through synergistic interaction [26,27]. For the reasons explained above, our hypothesis is that combining SC and BS could have a synergistic effect, improving growth performance and dietary energy utilization compared to when supplied separately. Therefore, a feeding trial was carried out with the aim of evaluating the effects of combining SC and BS in a high-energy diet offered to feedlot lambs during a long-term period (83 d) on growth performance, dietary energetics, carcass traits, shoulder tissue composition, and visceral organ mass.

## 2. Materials and Methods

All animal management procedures were in accordance with Mexico’s Federal Guidelines for Animal use and Care [28] and approved by the Ethics Committee of the School of Veterinary Medicine and Zootechnics, Autonomous University of Sinaloa (Approval Protocol #06072024).

The experiment was conducted at the Universidad Autónoma of Sinaloa Feedlot Lamb Research Unit, located in Culiacán (24°46′13″ N, 107°21′14″ W; 55 m.a.s.l.), México. Culiacán has a tropical climate. During the experiment, the average daily ambient temperature was 29.1 °C (the average minimum and maximum ambient temperatures were 26.4 and 33.8 °C, respectively), with an average relative humidity of 45.8% (the minimum and maximum relative humidity averaged 22.50 and 56.00%, respectively). The climatic conditions prevailing during the experiment were daily registered from a portable weather station (Thermo-hygrometer Avaly, Mod. DTH880, Mofeg S.A., Zapopan, Jal., Mexico). Calculating the temperature humidity index (THI) was using the following formula: THI = 0.81T + (RH/100) (T − 14.40) + 46.40, where T is the temperature expressed in degrees Celsius, and RH is the relative humidity [29].

### 2.1. Animals, Diet, and Experimental Design

Forty-eight Pelibuey × Katahdin lambs (98 ± 17 d age; initial weight = 20.25 ± 3.37 kg) were used in a randomized complete block design in a 2 × 2 factorial arrangement of treatments in order to evaluate the effects of combining two probiotics on growth performance, dietary energy use, carcass traits and relative weight of visceral organ mass. Lambs were received in the facilities 28 days before the start of the feeding trial and were subjected to the health management process, which consisted of deworming (Albendazole 10%, Animal Health and Welfare, México City, México), vitaminizing (injected 1 × 10^6^ IU vitamin A; Synt-ADE^®^, Fort Dodge, Animal Health, México City, México), and vaccination for Mannheimia haemolytica (One shot Pfizer, México City, México). Lambs were gradually adapted to the finishing diet for 3 weeks before the experiment started. At day 1 of the trial, lambs were weighed just prior to the morning meal (electronic scale; TORREY TIL/S: 107-2691, TORREY Electronics Inc., Houston, TX, USA), blocked by weight and ordered within weight groupings to 24 pens (2 lambs/pen, 6 pens/treatment). Pens were 6 m^2^ with overhead shade, soil floor (tucuruguay-sand mix), bucket waterers, and 1 m fence-line feed bunks. Lambs were fed with the same cracked corn-based finishing diet (Table 1) and treatments were tested as follows: (1) finishing diet without probiotic or prebiotic supplementation (Control), (2) finishing diet supplemented with 1.5 g autolyzed *Saccharomyces cerevisiae*/kg diet (SC; Hicell, dried autolyzed yeast contained 40% CP and a minimum of 60% β-glucans and 25% mannan-oligosaccharides, Biorigin, São Paulo, Brazil), (3) finishing diet supplemented with 1.5 g *Bacillus subtilis*/kg diet (BS; Clostat Dry, 2.0 × 10^11^ UFC/kg, Kemin Industries, Zapopan, Jal, México), and (4) finishing diet supplemented with 1.5 g SC plus 1.5 g BS/kg diet (SC + BS). The dosage of both probiotics was according to the manufacturer’s label recommendations, which in turn are derived from studies showing improvements in rumen fermentation, digestibility, or feed efficiency in finishing ruminants [10,30]. Dietary treatments were randomly assigned to pens within blocks. The probiotics of each treatment were premixed with 50 g of finely ground corn. To achieve the desired concentration in the final diet, the respective premix of each treatment was hand-weighed using a precision balance (Ohaus, mod. AS612, Pine Brook, NJ, USA) and premixed for 5 min with trace mineral salt before incorporation into complete mixed diets using a 2.5 m^3^ capacity paddle mixer (model 30910-7, Coyoacán, México). Lambs were provided fresh feed twice daily at 0800 and 1400 h, in which morning feed was offered constantly (300 g/lamb), while afternoon feed was offered ad libitum to allow for a feed residual of refusal of ~50 g/kg daily feed offering. Residual feed was daily collected around twenty minutes before 0:800 h each morning, weighed, and stored for subsequent DM analysis [31]. Dietary treatments were sampled weekly and composited for subsequent analysis. Composited samples were first oven-dried at 105 °C until no further weight loss (AOAC; 2000, method 930.15) to determine dry matter content [31]. Lambs were weighed just before the morning feeding on days 1 and 83, which was the final day. Live weights (LW) on day 1 were converted to shrunken body weight (SBW) by multiplying LW by 0.96 to account for the gastrointestinal fill [32]. All lambs were fasted for 18 h before recording the final LW, but drinking water was not withdrawn.

### 2.2. Calculations

The average daily gain was determined as the difference in initial SBW and final SBW divided by 83. Gain efficiency was estimated to be the ratio of ADG/DMI. In growth-performance trials, one way of more accurately evaluating dietary energy utilization efficiency is to compare observed-to-expected DMI and observed-to-expected dietary net energy (NE). The expected DMI estimation is performed based on observed ADG, average SBW, and NE values of the diet (Table 1): expected DMI, kg/d = (EM/2.04) + (EG/1.38), where EM (energy required for maintenance, Mcal/d) = 0.056 × SBW^0.75^, EG (energy gain, Mcal/d) = 0.276 × ADG × SBW^0.75^, and 2.04 and 1.38 are the NE_m_ and NE_g_ values contained in the basal diet according to the tabular values from NRC [33]. The coefficient (0.276) was taken from NRC [34], assuming a mature weight of 113 kg for Pelibuey × Katahdin male lambs.

The observed dietary NE was calculated using EM and EG values and the DMI observed during the experiment by means of the quadratic formula:

$$x=\frac{-b\pm \sqrt{b^{2}-4ac}}{2c}$$

where *x* = observed dietary NE_m_, Mcal/kg, *a* = −0.41 EM, *b* = 0.877 EM + 0.41 DMI + EG, and *c* = −0.877 DMI [35].

Then, the observed-to-expected dietary NE ratio is obtained by dividing the observed NE values by the expected NE content of the experimental diets. These expected values come from the ingredient composition of the basal diet, as shown in Table 1.

### 2.3. Carcass Characteristics, Whole Cuts, and Shoulder Tissue Composition Data

All the lambs were harvested at the final of the feeding trial (day 83) following the Mexican Federal Guidelines for Animal Use and Care. Lambs were stunned (captive bolt), exsanguinated, skinned, and the gastrointestinal organs were separated and weighed. Carcasses were chilled in a cooler at −2 to 1 °C for 24 h and fat thickness and LM surface area were measured following the procedures and methodology described by USDA [36]. The kidney, pelvic, and heart fat (KPH) was separated from the carcass and calculated as a percentage using the cold carcass weight (CCW) as a reference. Each carcass was cut in half. As recommended by the North American Meat Processors Association [37], the left side was prepared in wholesale cuts without trimming. The foresaddle provided the rack, breast, shoulder, and foreshank. From the hindsaddle, we removed the loins, flanks, and legs. We then recorded the weight of each cut. According to Luaces et al. [38], physical dissection was used to evaluate the composition of the shoulder’s tissue.

### 2.4. Visceral Mass Data

The tongue, esophagus, stomach (rumen, reticulum, omasum, and abomasum), pancreas, liver, gallbladder, small intestine (duodenum, jejunum, and ileum), and large intestine (cecum, colon, and rectum) were separated from the carcass and weighed. After washed and drained, all visceral organs were weighed again to determine empty weights. The empty body weight (EBW) was determined by deducting the difference between the full and washed digesta-free GIT. The ratio of the fresh tissue weight to the final EBW, which is the difference between the digesta weight and full live weight, is used to depict the organ mass. The sum of all visceral components, including digesta, including the stomach complex, small and large intestines, liver, lungs, and heart, was used to determine the total visceral mass. The digesta-free sum of the rumen, reticulum, omasum, and abomasum weights was used to estimate the stomach complex.

### 2.5. Statistical Analysis

The number of pen replicates (6) and animals (12) within treatments are enough to determine statistical differences on performance, carcass, and visceral mass variables of feedlot lamb. Based on power analysis and SD for measure, we had a power of 0.94 for detecting a 5% difference. All the data were tested for normality using the Shapiro–Wilk test. Data for growth performance, dietary energetics, carcass characteristics, shoulder muscle tissue, whole cuts, and visceral mass were analyzed as a randomized complete-block design in a 2 × 2 factorial arrangements of treatments [Factor A = *Saccharomyces cerevisiae* (SC), Factor B = *Bacillus subtilis* (BS), supplemented alone or in combination] by using the MIXED procedure of SAS software, Ver. 9.3 [39], considering the initial weight as the blocking criterion and pen as the experimental unit, with treatment and block as fixed effects and interaction block by treatment as random effect. The statistical model for the trial was as follows:

$$Y_{ijk}=\;\mu +\;W_{i}+\alpha_{j}+\beta_{k}+{\left(\alpha \beta \right)}_{jk}+\varepsilon_{ijk}$$

where $Y_{ijk}$ is the response variable, μ is the common experimental effect, $W_{i}$ is the block effect, $\alpha_{j}$ is the Factor A (SC) effect, $\beta_{k}$ is the Factor B (BS) effect, ${\left(\alpha \beta \right)}_{jk}$ is the interaction (SC × BS), and $\varepsilon_{ijk}$ is the random experimental Error $\varepsilon_{ijk\;}\sim \;IIN\;(0,\;\sigma_{\varepsilon }^{2})$.

Treatment effects were separated into the following orthogonal contrasts: (1) Control vs. SC supplementation; (2) Control vs. BS supplementation and (3) SC × BS interaction. For all variables measured, significance level was considered only when *p* ≤ 0.05. Given the low number of experimental units per treatment and subsamples within pens, trends were not desirable be considered.

## 3. Results

Through the trial, there were no losses or removals from experimental units due to death or illness.

Treatment effects on the variables evaluated are presented in Table 2, Table 3 and Table 4. In contradiction to our hypothesis, there were no synergistic (interaction) effects observed when combining SC + BS on any of the variables related to growth performance, dietary energy, tissue composition, whole cuts, or visceral mass measures. The responses observed for lambs that received the combination SC + BS were very similar to those that received SC supplementation alone, but greater than the control group and those that were supplemented with BS alone.

Lambs that were supplemented with BS alone showed a very similar response on dry matter intake (DMI, *p* = 0.41), average daily gain (ADG, *p* = 0.64), feed efficiency (*p* = 0.70), observed to expected dietary NE ratio (*p* = 0.52), carcass characteristics (*p* ≥ 0.08), shoulder tissue composition (*p* ≥ 0.32), and relative visceral organ mass (g/kg EBW, *p* ≥ 0.15) to non-supplemented lambs.

Compared to the Control group, lambs that received SC alone or in combination with BS showed greater average daily gain (12.0%, *p* = 0.03), gain efficiency (6.1%, *p* = 0.04) and observed-to expected dietary energy efficiency (5.5%, *p* = 0.04). Whereas, comparing SC alone vs. BS alone, lambs that received SC resulted in greater gain to feed ratio (6.0%, *p* = 0.05) and dietary energy utilization (5%, *p* = 0.04) than those that received BS.

Compared to BS and to non-supplemental lambs, supplemental SC and SC + BS increased hot carcass weight 8.8% (*p* = 0.04) without effects on the rest of variables evaluated (dressing percentage, LM area, and carcass fat depots) including the shoulder tissue composition and visceral organ mass.

## 4. Discussion

The average air temperature and relative humidity during the experiment were 29.1 ± 2.7 °C and 45.8 ± 8.1%, respectively. This translates to an average temperature humidity index (THI) value of 76.52 [29]. Silanikove [40] states that these environmental conditions are comfortable for well-adapted ruminants in high-heat environments, such as Pelibuey lambs and their crosses. Thus, the experiment took place under suitable climatic conditions.

### 4.1. Growth Performance and Dietary Energy

*SC plus BS supplementation*. Combinations of SC plus BS have been more commonly investigated in poultry [6]. The combination of SC plus *Lactobacillus* sp. has been more effective in improving spleen weight, and some immunological and intestinal parameters (villus height, muscular layer) in broilers that were challenged with mycotoxins [41]. Likewise, SC + BS combination has greatly improved growth performance in broilers compared to when they are supplemented only with SC or BS separately [6,42]; however, information about this combination in finishing ruminants is very limited. In denial of our hypothesis, there were no synergy (interaction) effects by combining SC + BS on any of the variables evaluated in the current experiment. The absence of interaction effects from the combination is surprising. Due to its characteristics, SC would theoretically limit its benefits only to the ruminal environment with very little impact on the lower tract [25], whereas, since BS is more resistant to surviving under adverse conditions, as well as being able to form robust protective biofilms against pathogenic agents [11], BS could be more effective at the lower tract level, complementing the benefits of S cerevisiae at this manner. Therefore, it is likely that combining both probiotics will improve their benefits for fermentation efficiency, microbial efficiency, and nutrient absorption in the rumen and postruminal tract. It is well-known that up to 80% of the digestible organic matter consumed is fermented in the rumen [43]. Thus, by increasing ruminal fermentation efficiency, the enhancements in growth and energy use from feed can be more noticeable. However, the cecum plays a crucial role in ruminant digestion by absorbing water and helping with the microbial digestion of leftover feedstuffs from the rumen. The fermentable substances that reach the cecum are different from those in the rumen. This difference can lead to changes in the composition or structure of the microbiota in these two sites. From an energetic perspective, the energy contributions from postruminal fermentation are significant. The cecum serves as an additional energy source, accounting for up to 8.6% of metabolizable energy intake [44]. Additionally, around 10% of the methane produced by ruminants comes from microbial activity in the cecum [45]. Therefore, improvements in fermentation processes at this level can enhance productive efficiency and decrease enteric methane production in ruminants. It is widely known that the site (ruminal or postruminal) and extent of OM digestion are affected by the concentrate level in the diet and by the level of intake. This is more relevant if it is considered that the passage of solid digesta is faster in lambs than in cattle [46]. In such a way that postruminal fermentation improvements may be more interesting in lambs consuming high-concentrate diets [47]. Nevertheless, the combination SC + BS did not improve productive response compared with lambs supplemented with SC alone. It is important to note, as is discussed below, that in the current experiment, lambs that were supplemented with BS alone did not show any improvement compared to non-supplemented lambs. This absence of effects in lambs supplemented with BS could partially explain why the combination failed to show the expected synergistic effect in the current experiment.

*BS supplementation*. *Bacillus subtilis* has been effectively used as a feed additive to improve growth performance in pigs [12,13,14] and poultry [15,16,17]. The positive effects on growth seem to show mainly when pigs and broilers receive BS supplements under disease challenges. For ruminants in feedlot systems, the response to BS supplementation does not appear to be the same as in non-ruminant species. The precise reason for explaining why finishing cattle that have been supplemented with BS do not have the same positive responses on productive parameters as non-ruminant species is, for now, beyond our reach. Mielich-Süss and Lopez [11] attribute to BS primarily the protective action against enteric pathogens; however, it has also been associated with changes that promote fermentative efficiency through the production of different kinds of enzymes [48] and changes in rumen fermentation that can lead to a rise in cellulolytic bacteria [49], which may boost cattle productivity. Nevertheless, in line with our results, the lack of positive effects of BS supplementation has been previously reported. In this regard, lambs that were fed during 56 days with a finishing diet very similar to the one used in the current experiment and which was supplemented with 2 g BS (2.2 × 10^8^ CFU)/kg diet, did not show improvements on ADG, feed efficiency nor dietary energy utilization when it was supplemented alone, but enhanced growth performance parameters only when was supplemented in combination with antibiotic monensin [20]. Feedlot cattle that received 2 g of BS daily during a short period, specifically 35 days on a finishing diet with 20% corn silage as the only forage source and an energy content of 1.97 Mcal NE_m_/kg diet, did not show differences in growth performance, dietary energy use, or carcass traits compared to non-supplemented cattle. Similarly, long-term supplementation over 258 days with 28 g of a mix of BS and *Lactobacillus plantarum* for cattle fattening in favorable conditions resulted in comparable growth performance, gain efficiency, dietary energy use, and carcass traits as seen in unsupplemented cattle [50]. In contrast, a preparation consisting of spore-forming bacteria, *Bacillus subtilis* and *Bacillus licheniformis*, did improve weight gain, but did not enhance feed efficiency in growing lambs [51]. Based on existing reports and our results, it appears that BS supplementation alone in healthy lambs or cattle finished under favorable environmental conditions (without stress from adverse weather or disease) does not provide any productivity benefits. Further research on BS supplementation on postruminal fermentation in feedlot lambs under various environmental conditions could provide better insights into the potential of BS as a feed additive in finishing diets.

*SC supplementation*. Autolyzed yeasts are made by breaking down yeast cells, specifically *Saccharomyces cerevisiae*, using their own enzymes. This process, called autolysis, releases amino acids like lysine and glutamate, peptides, mannan oligosaccharides, glucans, and nucleotides [52]. These components provide important prebiotic properties. The main effects of yeast supplements, whether live or dried, are that they change intestinal flora through a competitive process with the less beneficial bacteria of the GIT, altering rumen fermentation and promoting GIT health, analogous to some antibiotics used as feed additives [4,53]. These effects have been associated with increased animal growth rate and/or feed efficiency in cattle [8] and in lambs [9,10,54].

However, more studies are still needed, especially with small ruminants (sheep and goats), to shed more light on the specific effects of SC and its mode of action on lamb performance [55]. Although there are inconsistencies regarding the benefits of SC supplementation on ruminant productivity, these appear to depend primarily on diet composition (i.e., concentrate: forage ratio) and dosage. In this context, high-concentration diets that compromise ruminal function by promoting low ruminal pH (<6.0) can be partially overcome with YC supplementation [56,57]. The basal diet used in this experiment contained 88% concentrates. Given this diet composition, according to NRC [58], the predicted ruminal pH of lambs was 5.76. Therefore, the presence of SC in the diet may have contributed to improved ruminal function under these conditions in this trial. The other condition concerns the dose used. Doses of live yeast greater than 5.5 CFU have been shown to have a consistent positive effect on sheep productivity [59,60,61]. However, autolyzed yeast consists only of cell wall components, and the dosage is specified as autolyzed yeast ingested/day or per kg of diet. Although benefits on productive performance have been observed with dosages ranging from 0.6 to 12 g SC/kg diet in some studies [62,63], other studies failed to show improvements in productive performance when ruminants were supplemented at these same dosage levels. For example, supplementation of autolyzed SC at a rate of 3 g/kg of diet improved feed efficiency by 6.9% and dietary net energy by 5.9% in lambs finished on a diet containing a 90:10 concentrates-to-forage ratio [64], whereas in another study, supplementation of autolyzed SC yeast in the diet of Dorper × Santa Ines lambs finished in a feedlot for 70 days did not significantly improve overall animal performance [65]. Those researchers concluded that adding 5 g/animal/day of autolyzed yeast to a total mixed ration does not improve animal performance, including the final body weight, DM intake, average daily gain, and feed conversion ratio, which remain similar to those of control groups. The debate over the best dosage may stem from the product’s nature. Autolyzed yeast has different compositions depending on its production method. Typically, autolyzed yeast from *Saccharomyces cerevisiae* has β-glucans ranging from 29% to 64% and about 31% mannan-oligosaccharides [66]. It is important to note that yeasts come in different forms; therefore, the technology to produce them is essential to maintain their active concentration and determine how they act, whether they are stronger or weaker. Therefore, the optimal dosage should be determined based on the concentrations of the product’s components (i.e., CP, amino acids, and glucans).

### 4.2. Carcass Traits and Visceral Mass Responses

According to previous studies, probiotic or prebiotic supplementation did not significantly impact carcass characteristics [67,68], tissue composition [69], or wholesale cuts [64,70] in feedlot lambs. The increase in hot carcass weight (HCW) among lambs supplemented with SC or a combination of supplements in this experiment is expected. This is because SC alone or in combination increased the rate of weight gain compared to the control group, leading to a heavier final weight at slaughter.

In accordance with Belewu and Jimoh [71] and Raghebian et al. [72], there were no treatment effects on the stomach complex, liver, heart, kidney, and lung mass. Numerous studies indicate that adding probiotics and prebiotics during the fattening phase generally does not significantly affect carcass traits, tissue composition, or visceral mass in ruminants [8,64,73,74].

Probiotics have been shown to reduce the thickness of intestinal walls and inflammation in mammals [75,76] and birds [77] by blocking pro-inflammatory factors and triggering protective proteins in intestinal cells. While there is limited information on the effects of BS and SC, or their combination, on intestinal mass in lambs fed high-energy diets, similar to our results, some studies found that adding BS, SC, and live yeasts, whether used alone or together, did not change the relative intestinal mass in lambs on high-energy diets [20,64].

## 5. Conclusions

Under the conditions of this experiment, we concluded that autolyzed SC improves growth performance and dietary energy in finishing lambs without altering carcass traits or composition. Contrary to our expectations, combining autolyzed SC with BS did not enhance the response compared to autolyzed SC alone. This could occur because the magnitude of benefits of BS at the postruminal level is not similar to what happens in non-ruminant species. In fact, in the current study, including 1.5 g BS/kg diet over a long-term (83-day) period did not improve performance or carcass traits in finishing lambs. Further research on the effect of BS supplementation on postruminal fermentation in feedlot lambs, as well as studies under different environmental conditions, could provide clearer information on the potential of BS as a feed additive in ruminant finishing diets.

## Figures and Tables

**Table 1:** Composition of basal diet and treatments.

	Treatments			
Item	Control	SC	BS	SC + BS
Ingredient composition (%)				
Sudangrass hay	12.00	12.00	12.00	12.00
Cracked corn	57.00	57.00	57.00	57.00
Soybean meal	14.00	14.00	14.00	14.00
*Saccharomyces cerevisiae* ^1^	0.00	+++	0.00	0.00
*Bacillus subtilis* ^2^	0.00	0.00	+++	0.00
SC plus BS	0.00	0.00	0.00	+++
Molasses cane	8.00	8.00	8.00	8.00
Yellow grease	3.50	3.50	3.50	3.50
Zeolite clay	3.00	3.00	3.00	3.00
Trace protein-mineral salt ^3^	2.50	2.50	2.50	2.50
Chemical composition (%DM basis) ^4^				
Crude protein	14.42	14.42	14.42	14.42
Neutral detergent fiber	16.20	16.20	16.20	16.20
Ether extract	6.76	6.76	6.76	6.76
Calculated net energy (Mcal/kg)				
Maintenance	2.04	2.04	2.04	2.04
Gain	1.38	1.38	1.38	1.38

+++ Indicate that the additive is included. SC = *Saccharomyces cerevisiae*; BS = *Bacillus subtilis.* ^1^ Diet supplemented with 1.5 g autolyzed *Saccharomyces cerevisiae*/kg diet (dried autolyzed yeast) contained 40%CP and a minimum of 60% β-glucans and 25% mannan-oligosaccharides. ^2^ Diet supplemented with 1.5 g *Bacillus subtilis*/kg diet (2.0 × 10^11^ UFC/kg). ^3^ Mineral premix contained: Crude protein 72.8%, Calcium, 20%; CoSO_4_, 0.010%; CuSO_4_, 0.15%; FeSO_4_, 0.528%; ZnO, 0.111%; MnSO_4_, 0.160%; KI, 0.007%; and NaCl, 13.7%. ^4^ Calculated based on tabular net energy (NE) and nutrient composition values for individual feed ingredients [33].

**Table 2:** Treatment effects on growth performance and observed dietary energy of lambs after fattening period of 83 days.

	Feed		Additives ^1^			*p*-Value		
Item	Control	SC	BS	SC + BS	SEM	SC	BS	SC × BS
Live weight, kg								
Initial	20.17	20.22	20.30	20.30	0.155	0.87	0.51	0.88
Final	40.48	43.25	41.72	42.90	0.444	0.03	0.29	0.62
Weight gain, kg/d	0.245	0.277	0.258	0.272	0.007	0.04	0.36	0.64
DM intake, kg	1.002	1.059	1.043	1.056	0.029	0.12	0.39	0.40
Gain to feed ratio	0.244	0.262	0.247	0.258	0.003	0.04	0.59	0.70
Diet energy, Mcal/kg								
Maintenance	2.02	2.12	2.03	2.10	0.012	0.05	0.54	0.52
Gain	1.36	1.45	1.37	1.43	0.010	0.05	0.54	0.52
Observed-to-expected diet NE								
Maintenance	0.99	1.04	1.00	1.04	0.008	0.05	0.54	0.52
Gain	0.99	1.05	1.00	1.03	0.009	0.05	0.54	0.52

^1^ SC = Diet supplemented with 1.5 g autolyzed *Saccharomyces cerevisiae*/kg diet (dried autolyzed yeast) contained 40% CP and a minimum of 60% β-glucans and 25% mannan-oligosaccharides. BS = Diet supplemented with 1.5 g *Bacillus subtilis*/kg diet (2.0 × 10^11^ UFC/kg).

**Table 3:** Treatment effects on carcass characteristics, shoulder tissue composition, and whole cuts of lambs after fattening period of 83 days.

	Feed		Additives ^1^			*p*-Value		
Item	Control	SC	BS	SC + BS	SEM	SC	BS	SC × BS
Hot carcass weight, kg	23.17	25.42	23.90	25.01	0.406	0.04	0.33	0.80
Dressing percentage	57.17	58.77	57.29	58.28	0.449	0.19	0.73	0.87
Cold carcass weight, kg	22.91	25.15	23.61	24.78	0.404	0.04	0.35	0.82
LM area, cm^2^	13.79	13.96	13.95	14.01	0.265	0.81	0.85	0.82
Fat thickness, cm	2.42	2.30	2.22	2.25	0.125	0.68	0.36	0.45
KPH, %	2.71	3.33	3.07	2.94	0.148	0.36	0.75	0.11
Shoulder tissue composition,%								
Lean	66.49	66.58	66.88	66.38	0.612	0.48	0.47	0.76
Fat	14.49	15.37	14.61	13.77	0.592	0.98	0.39	0.32
Bone	19.02	19.05	18.51	19.85	0.455	0.35	0.89	0.37
Lean: fat ratio	4.76	4.42	4.70	4.84	0.239	0.77	0.60	0.49
Bone: fat ratio	3.50	3.61	3.50	3.37	0.141	0.40	0.98	0.47
Whole cuts (as % of CCW)								
Forequarter	40.55	41.28	39.60	37.46	1.398	0.73	0.25	0.48
Neck	8.44	8.01	7.99	8.62	0.376	0.80	0.83	0.19
Shoulder IMPS206	8.85	8.03	7.69	8.05	0.316	0.63	0.22	0.21
Shoulder IMPS207	14.40	14.37	14.38	14.34	0.271	0.92	0.95	0.99
Rack IMPS204	7.19	7.26	6.92	7.20	0.122	0.33	0.37	0.54
Breast IMPS209	4.38	5.29	4.06	4.32	0.293	0.18	0.15	0.45
Ribs IMPS209A	6.36	6.57	6.40	6.50	0.206	0.14	0.74	0.62
Hindquarter	36.49	36.68	34.11	37.37	1.343	0.42	0.65	0.45
Loin IMPS231	6.97	6.98	7.00	7.04	0.105	0.86	0.77	0.95
Flank IMPS232	5.87	6.32	6.02	6.26	0.142	0.12	0.79	0.60
Leg IMPS233	23.61	23.21	24.39	23.81	0.332	0.32	0.17	0.86

LM = longissimus muscle; KPH = kidney-pelvic-hearth fat; CCW = cold carcass weight. ^1^ SC = Diet supplemented with 1.5 g autolyzed *Saccharomyces cerevisiae*/kg diet (dried autolyzed yeast contained 40% CP and a minimum of 60% β-glucans and 25% mannan-oligosaccharides. BS = Diet supplemented with 1.5 g *Bacillus subtilis*/kg diet (2.0 × 10^11^ UFC/kg).

**Table 4:** Treatment effects on relative visceral organ mass of lambs after fattening period of 83 days.

	Feed		Additives ^1^			*p*-Value		
Item	Control	SC	BS	SC + BS	SEM	SC	BS	SC × BS
EBW, % of full weight	90.20	91.80	90.90	90.96	0.406	0.11	0.79	0.72
Organs, g/kg EBW								
Stomach complex	29.90	29.71	29.00	30.62	0.780	0.37	0.15	0.91
Intestines	39.39	38.22	39.31	38.63	0.831	0.58	0.72	0.83
Heart + lungs	20.79	20.81	20.45	20.31	0.580	0.41	0.84	0.87
Liver + spleen	20.44	21.87	20.40	20.31	0.659	0.24	0.21	0.82
Kidney	2.85	2.71	2.88	2.93	0.093	0.94	0.12	0.15
Visceral fat	35.17	37.28	38.04	38.31	1.982	0.68	0.50	0.75

EBW = empty body weight. ^1^ SC = Diet supplemented with 1.5 g autolyzed *Saccharomyces cerevisiae*/kg diet (dried autolyzed yeast contained 40%CP and a minimum of 60% β-glucans and 25% mannan-oligosaccharides. BS = Diet supplemented with 1.5 g *Bacillus subtilis*/kg diet (2.0 × 10^11^ UFC/kg).

## Data Availability

The data that support the findings of this study are available from the corresponding authors upon reasonable request.
